# Crystal structure of (*E*)-3-(5-bromo-2-hydroxy­phen­yl)acryl­aldehyde

**DOI:** 10.1107/S1600536814023708

**Published:** 2014-10-31

**Authors:** Sung-Gon Kim

**Affiliations:** a Department of Chemistry, Kyonggi University, 154-42, Gwanggyosan-ro, Yeongtong-gu, Suwon 443-760, Republic of Korea

**Keywords:** crystal structure, *trans* configuration, vinyl­aldehyde group, hydrogen bonding, three-dimensional supra­molecular network 2-hy­droxy­cinnamaldehydes

## Abstract

The title compound, C_9_H_7_BrO_2_, displays a *trans* configuration with respect to the C=C double bond and is essentially planar [maximum deviation from the least-squares plane through all non-H atoms = 0.056 (4) Å]. The vinyl­aldehyde group adopts an extended conformation wih a C—C—C—C torsion angle of 179.7 (4)°. In the crystal, mol­ecules are linked by classical O—H⋯O and weak C—H⋯O hydrogen bonds into a three-dimensional supra­molecular network.

## Related literature   

For the synthesis of 2-hy­droxy­cinnamaldehydes, see: Kim *et al.* (2004 ▶); Zeiter & Rose (2009 ▶). For their biological activity, see: Kwon *et al.* (1996 ▶); Lee *et al.* (1999 ▶); Ka *et al.* (2003 ▶); Gan *et al.* (2009 ▶); Han *et al.* (2011 ▶). For their synthetic applications, see: Cabrera *et al.* (2008 ▶); Zu *et al.* (2009 ▶); Choi & Kim (2010 ▶); Lee & Kim (2011 ▶); Lee *et al.* (2011 ▶). For related structures, see: Kang & Kim (2013 ▶).
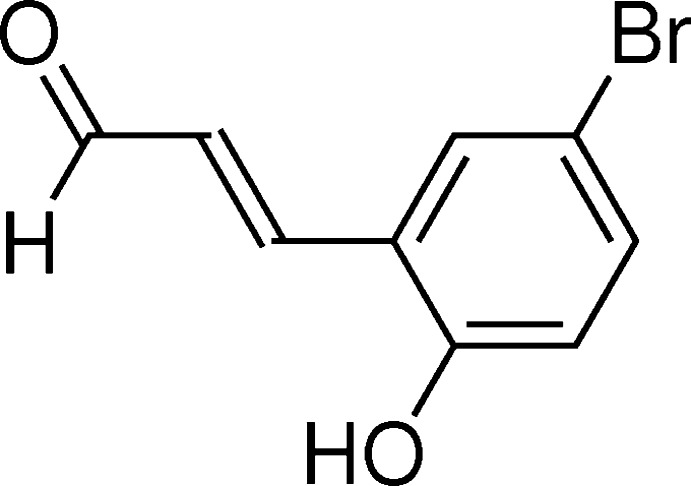


## Experimental   

### Crystal data  

C_9_H_7_BrO_2_
*M* *_r_* = 227.06
Orthorhombic, 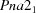

*a* = 12.1230 (11) Å
*b* = 15.0901 (14) Å
*c* = 4.8763 (4) Å
*V* = 892.06 (14) Å^3^
*Z* = 4
Mo *K*α radiation
μ = 4.56 mm^−1^
*T* = 200 K
0.41 × 0.35 × 0.15 mm

### Data collection  

Bruker SMART CCD area-detector diffractometer
Absorption correction: multi-scan (*SADABS*; Bruker, 2007 ▶) *T*_min_ = 0.256, *T*_max_ = 0.548
6031 measured reflections
2052 independent reflections
1683 reflections with *I* > 2σ(*I*)
*R*_int_ = 0.024

### Refinement  

*R*[*F* ^2^ > 2σ(*F* ^2^)] = 0.028
*wR*(*F* ^2^) = 0.086
*S* = 1.16
2052 reflections
110 parameters
1 restraint
H-atom parameters constrained
Δρ_max_ = 0.48 e Å^−3^
Δρ_min_ = −0.58 e Å^−3^
Absolute structure: Flack (1983 ▶)
Absolute structure parameter: 0.021 (19)

### 

Data collection: *SMART* (Bruker, 2007 ▶); cell refinement: *SAINT* (Bruker, 2007 ▶); data reduction: *SAINT*; program(s) used to solve structure: *SHELXTL* (Sheldrick, 2008 ▶); program(s) used to refine structure: *SHELXTL*; molecular graphics: *ORTEP-3 for Windows* (Farrugia, 2012 ▶); software used to prepare material for publication: *SHELXTL* and *publCIF* (Westrip, 2010 ▶).

## Supplementary Material

Crystal structure: contains datablock(s) I, global. DOI: 10.1107/S1600536814023708/gw2149sup1.cif

Structure factors: contains datablock(s) I. DOI: 10.1107/S1600536814023708/gw2149Isup2.hkl

Supporting information file. DOI: 10.1107/S1600536814023708/gw2149Isup3.cml

**Fig. 1.**
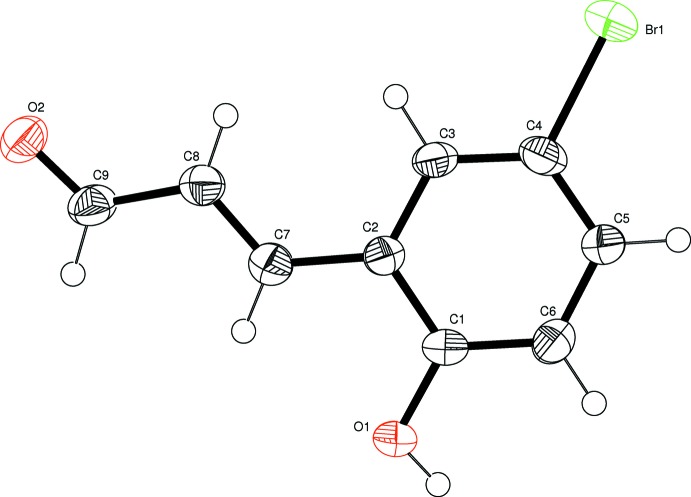
A view of the molecular structure of the title compound, showing the atom-numbering scheme. Displacement ellipsoids are drawn at the 50% probability level.

. DOI: 10.1107/S1600536814023708/gw2149fig1.tif
A view of the mol­ecular structure of the title compound, showing the atom-numbering scheme. Displacement ellipsoids are drawn at the 50% probability level.

**Fig. 2.**
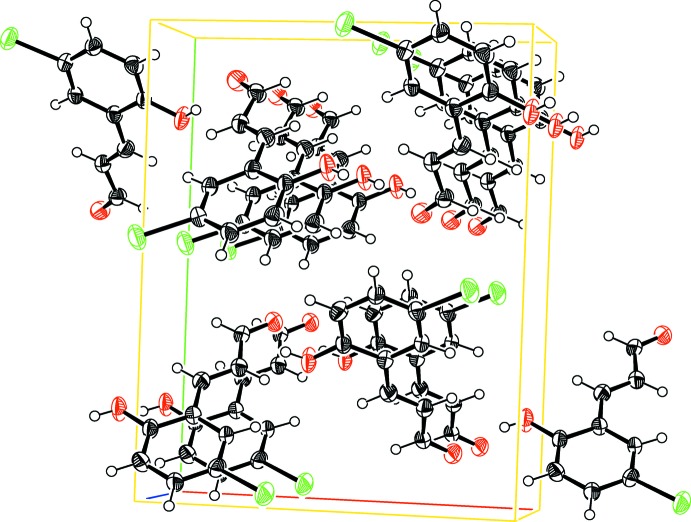
A partial view of the crystal packing of the title compound. Hydrogen atoms have been omitted for clarity.

. DOI: 10.1107/S1600536814023708/gw2149fig2.tif
A partial view of the crystal packing of the title compound. Hydrogen atoms have been omitted for clarity.

CCDC reference: 1031276

Additional supporting information:  crystallographic information; 3D view; checkCIF report

## Figures and Tables

**Table 1:** Hydrogen-bond geometry (, )

*D*H*A*	*D*H	H*A*	*D* *A*	*D*H*A*
O1H1O2^i^	0.84	1.89	2.693(5)	160
C5H5O2^ii^	0.95	2.35	3.267(6)	162

Symmetry codes: (i) 
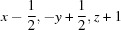
; (ii) 
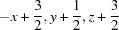
.

## supplementary crystallographic information

### S2. Structural commentary

For related structures, see: Kang *et al.* (2013). 2-Hy­droxy­cinnamaldehyde, isolated from the stern bark of *cinnamonum cassia*, and its synthetic derivatives have been shown to inhibit on farnesyl protein transferase in vitro as well as angiogenesis. (Kwon *et al.* 1996; Lee *et al.* 1999; Ka *et al.* 2003). Andrecently they also have been reported to have anti­tumor effects against various human tumor cells in vitro and in vivo (Gan *et al.* 2009; Han *et al.* 2011).In view of these potential applications and in continuation of our work, the structure of the title compound has been carried out and the results are presented here.

X-ray analysis confirms the molecular structure and atom connectivity as illustrated in Fig. 1. The title compound is essentially planar. The C==C double bond is in an *E* conformation and the vinyl­aldehyde groups adopt extended conformations as can be seen from the torsion angles C2—C7—C8—C9 = 179.7 (4)°. In the crystal, the molecules are linked by classic O—H···O hydrogen bond and weak C—H···O hydrogen bonds (Table 1).

### S3. Synthesis and crystallization

5-Bromo-2-hy­droxy­benzaldehyde (5.0 mmol, 1.01 g) and (tri­phenyl­phospho­ranyl­idene)acetaldehyde (6.0 mmol, 1.83 g) were dissolved in benzene (50 ml). After stirring for 6 h, solvent was evaporated. Purification by silica gel chromatography was afforded the title compound. Crystals suitable for X-ray analysis were obtained by recryatallization from an n-hexane/CH_2_Cl_2_ solution..

### S4. Refinement

All H atoms were positioned geometrically, (O—H = 0.84 A ° and C—H = 0.95–0.96 Å) and constrained to ride on their parent atoms, with *U*_iso_(H) = *xU*eq(C), where *x* = 1.2 for all other H atoms.

### Crystal data

**Table:**

C_9_H_7_BrO_2_	*F*(000) = 448
*M**_r_* = 227.06	*D*_x_ = 1.691 Mg m^−^^3^
Orthorhombic, *P**n**a*2_1_	Mo *K*α radiation, λ = 0.71073 Å
Hall symbol: P 2c -2n	Cell parameters from 3701 reflections
*a* = 12.1230 (11) Å	θ = 2.7–28.2°
*b* = 15.0901 (14) Å	µ = 4.56 mm^−^^1^
*c* = 4.8763 (4) Å	*T* = 200 K
*V* = 892.06 (14) Å^3^	Block, pale yellow
*Z* = 4	0.41 × 0.35 × 0.15 mm

### Data collection

**Table:**

Bruker SMART CCD area-detector diffractometer	2052 independent reflections
Radiation source: fine-focus sealed tube	1683 reflections with *I* > 2σ(*I*)
Graphite monochromator	*R*_int_ = 0.024
phi and ω scans	θ_max_ = 28.3°, θ_min_ = 2.2°
Absorption correction: multi-scan (*SADABS*; Bruker, 2007)	*h* = −16→13
*T*_min_ = 0.256, *T*_max_ = 0.548	*k* = −20→19
6031 measured reflections	*l* = −6→5

### Refinement

**Table:**

Refinement on *F*^2^	Secondary atom site location: difference Fourier map
Least-squares matrix: full	Hydrogen site location: inferred from neighbouring sites
*R*[*F*^2^ > 2σ(*F*^2^)] = 0.028	H-atom parameters constrained
*wR*(*F*^2^) = 0.086	*w* = 1/[σ^2^(*F*_o_^2^) + (0.*P*)^2^ + 1.2584*P*] where *P* = (*F*_o_^2^ + 2*F*_c_^2^)/3
*S* = 1.16	(Δ/σ)_max_ = 0.001
2052 reflections	Δρ_max_ = 0.48 e Å^−^^3^
110 parameters	Δρ_min_ = −0.58 e Å^−^^3^
1 restraint	Absolute structure: Flack (1983)
Primary atom site location: structure-invariant direct methods	Absolute structure parameter: 0.021 (19)

### Special details

**Table:**

Geometry. All e.s.d.'s (except the e.s.d. in the dihedral angle between two l.s. planes) are estimated using the full covariance matrix. The cell e.s.d.'s are taken into account individually in the estimation of e.s.d.'s in distances, angles and torsion angles; correlations between e.s.d.'s in cell parameters are only used when they are defined by crystal symmetry. An approximate (isotropic) treatment of cell e.s.d.'s is used for estimating e.s.d.'s involving l.s. planes.
Refinement. Refinement of *F*^2^ against ALL reflections. The weighted *R*-factor *wR* and goodness of fit *S* are based on *F*^2^, conventional *R*-factors *R* are based on *F*, with *F* set to zero for negative *F*^2^. The threshold expression of *F*^2^ > σ(*F*^2^) is used only for calculating *R*-factors(gt) *etc*. and is not relevant to the choice of reflections for refinement. *R*-factors based on *F*^2^ are statistically about twice as large as those based on *F*, and *R*- factors based on ALL data will be even larger.

### Fractional atomic coordinates and isotropic or equivalent isotropic displacement parameters (Å^2^)

**Table:**

	*x*	*y*	*z*	*U*_iso_*/*U*_eq_	
C1	0.5560 (4)	0.3452 (3)	0.1499 (9)	0.0325 (9)	
O1	0.4596 (3)	0.3084 (2)	0.0686 (7)	0.0452 (8)	
H1	0.4091	0.3236	0.1766	0.068*	
C2	0.6537 (4)	0.3149 (3)	0.0237 (8)	0.0295 (9)	
C3	0.7531 (3)	0.3521 (3)	0.1065 (9)	0.0305 (9)	
H3	0.8200	0.3336	0.0229	0.037*	
C4	0.7550 (3)	0.4153 (3)	0.3081 (10)	0.0352 (10)	
Br1	0.89425 (3)	0.45978 (3)	0.4305 (2)	0.04971 (16)	
C5	0.6611 (3)	0.4457 (2)	0.4299 (17)	0.0350 (8)	
H5	0.6645	0.4905	0.5663	0.042*	
C6	0.5603 (4)	0.4099 (3)	0.3509 (8)	0.0359 (11)	
H6	0.4942	0.4299	0.4351	0.043*	
C7	0.6470 (4)	0.2463 (3)	−0.1865 (9)	0.0350 (9)	
H7	0.5764	0.2211	−0.2199	0.042*	
C8	0.7312 (4)	0.2157 (3)	−0.3365 (10)	0.0339 (9)	
H8	0.8032	0.2391	−0.3108	0.041*	
C9	0.7127 (3)	0.1470 (3)	−0.5372 (13)	0.0368 (10)	
H9	0.6403	0.1234	−0.5524	0.044*	
O2	0.7830 (3)	0.1175 (2)	−0.6872 (7)	0.0426 (8)	

### Atomic displacement parameters (Å^2^)

**Table:**

	*U*^11^	*U*^22^	*U*^33^	*U*^12^	*U*^13^	*U*^23^
C1	0.022 (2)	0.038 (2)	0.038 (2)	−0.0013 (17)	0.0002 (17)	−0.0013 (19)
O1	0.0208 (16)	0.062 (2)	0.053 (2)	−0.0040 (15)	0.0017 (14)	−0.0239 (17)
C2	0.026 (2)	0.033 (2)	0.029 (2)	0.0007 (16)	0.0008 (15)	−0.0015 (16)
C3	0.021 (2)	0.032 (2)	0.039 (2)	0.0016 (16)	0.0024 (17)	0.0006 (18)
C4	0.026 (2)	0.035 (2)	0.045 (2)	−0.0046 (17)	−0.0061 (18)	−0.001 (2)
Br1	0.0290 (2)	0.0552 (3)	0.0649 (3)	−0.00854 (18)	−0.0047 (3)	−0.0132 (3)
C5	0.0267 (18)	0.0353 (18)	0.043 (2)	−0.0007 (14)	0.002 (4)	−0.007 (3)
C6	0.034 (2)	0.038 (2)	0.036 (3)	0.0056 (17)	0.0033 (17)	−0.0051 (18)
C7	0.031 (2)	0.035 (2)	0.039 (2)	−0.0026 (18)	0.0010 (19)	−0.0035 (19)
C8	0.028 (2)	0.035 (2)	0.039 (2)	0.0007 (18)	−0.0009 (19)	−0.0018 (18)
C9	0.029 (2)	0.0342 (19)	0.047 (3)	−0.0032 (15)	0.008 (2)	−0.002 (2)
O2	0.0374 (19)	0.0407 (17)	0.050 (2)	0.0004 (14)	0.0109 (14)	−0.0110 (15)

### Geometric parameters (Å, º)

**Table:**

C1—O1	1.353 (5)	C5—C6	1.391 (6)
C1—C6	1.384 (6)	C5—H5	0.9500
C1—C2	1.411 (6)	C6—H6	0.9500
O1—H1	0.8400	C7—C8	1.339 (6)
C2—C3	1.389 (6)	C7—H7	0.9500
C2—C7	1.459 (6)	C8—C9	1.443 (7)
C3—C4	1.370 (6)	C8—H8	0.9500
C3—H3	0.9500	C9—O2	1.208 (6)
C4—C5	1.363 (6)	C9—H9	0.9500
C4—Br1	1.913 (4)		
			
O1—C1—C6	122.0 (4)	C4—C5—H5	120.6
O1—C1—C2	117.6 (4)	C6—C5—H5	120.6
C6—C1—C2	120.4 (4)	C1—C6—C5	120.2 (4)
C1—O1—H1	109.5	C1—C6—H6	119.9
C3—C2—C1	118.1 (4)	C5—C6—H6	119.9
C3—C2—C7	122.6 (4)	C8—C7—C2	125.9 (4)
C1—C2—C7	119.3 (4)	C8—C7—H7	117.0
C4—C3—C2	120.3 (4)	C2—C7—H7	117.0
C4—C3—H3	119.9	C7—C8—C9	120.0 (4)
C2—C3—H3	119.9	C7—C8—H8	120.0
C5—C4—C3	122.2 (4)	C9—C8—H8	120.0
C5—C4—Br1	118.9 (4)	O2—C9—C8	124.5 (4)
C3—C4—Br1	118.9 (3)	O2—C9—H9	117.8
C4—C5—C6	118.9 (5)	C8—C9—H9	117.8
			
O1—C1—C2—C3	−179.8 (4)	Br1—C4—C5—C6	−176.7 (4)
C6—C1—C2—C3	−0.2 (6)	O1—C1—C6—C5	179.6 (5)
O1—C1—C2—C7	0.1 (6)	C2—C1—C6—C5	0.1 (7)
C6—C1—C2—C7	179.7 (4)	C4—C5—C6—C1	−0.7 (8)
C1—C2—C3—C4	1.0 (6)	C3—C2—C7—C8	−5.0 (7)
C7—C2—C3—C4	−178.9 (4)	C1—C2—C7—C8	175.1 (4)
C2—C3—C4—C5	−1.7 (7)	C2—C7—C8—C9	179.7 (4)
C2—C3—C4—Br1	176.5 (3)	C7—C8—C9—O2	177.5 (5)
C3—C4—C5—C6	1.5 (8)		

### Hydrogen-bond geometry (Å, º)

**Table:**

*D*—H···*A*	*D*—H	H···*A*	*D*···*A*	*D*—H···*A*
O1—H1···O2^i^	0.84	1.89	2.693 (5)	160
C5—H5···O2^ii^	0.95	2.35	3.267 (6)	162

Symmetry codes: (i) *x*−1/2, −*y*+1/2, *z*+1; (ii) −*x*+3/2, *y*+1/2, *z*+3/2.
