# Supplementary figures and images for: Crystal structure of (E)-3-(5-bromo-2-hydroxy­phen­yl)acryl­aldehyde
Source: Acta Crystallogr Sect E Struct Rep Online. 2014 Oct 31;70(Pt 11):o1219–20. doi: 10.1107/S1600536814023708 (PMC4257279; doi:10.1107/S1600536814023708)

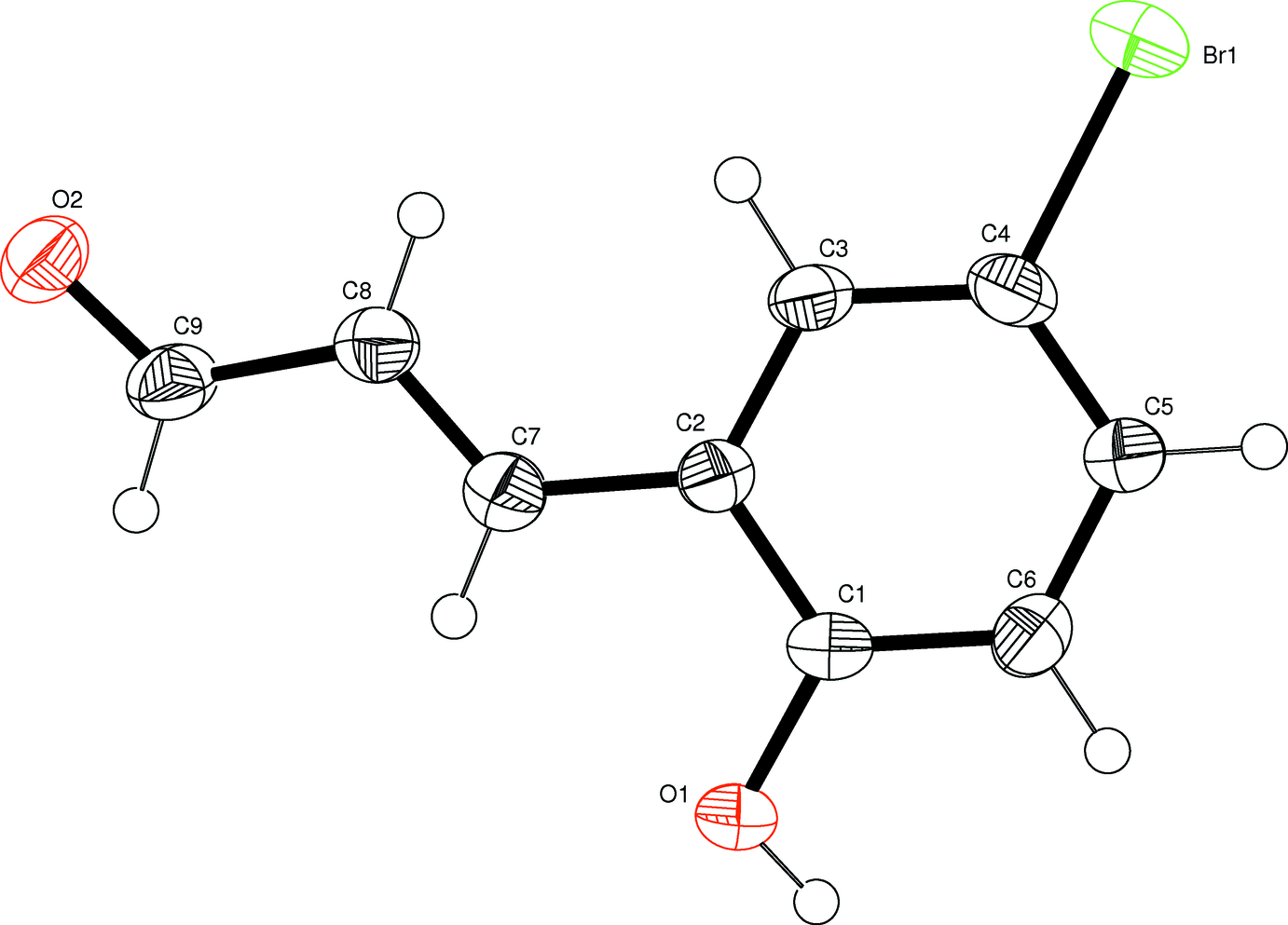

Supplement: Supplementary file 4 [file e-70-o1219-fig1.tif]

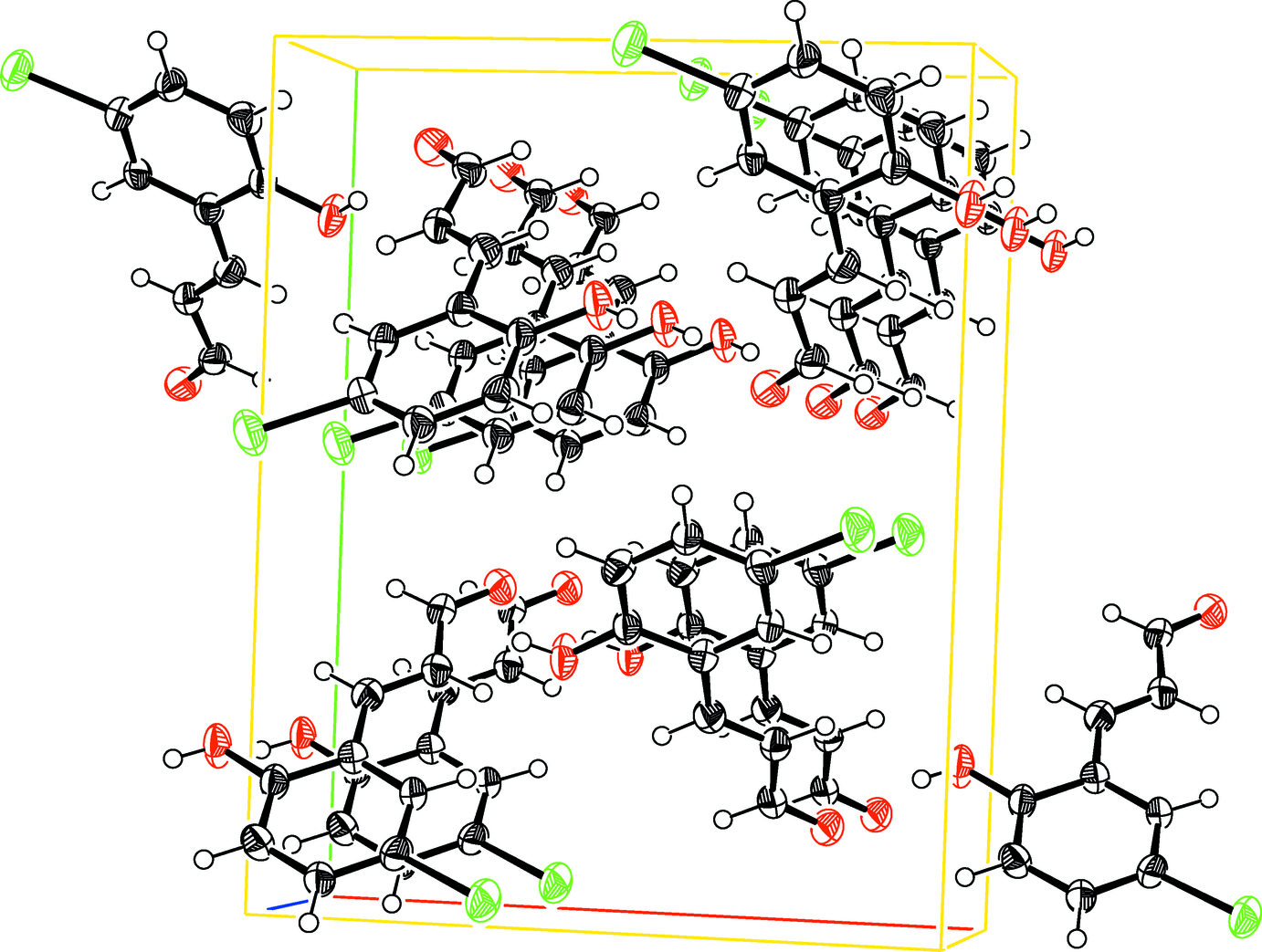

Supplement: Supplementary file 5 [file e-70-o1219-fig2.tif]
